# CSF YKL‐40 demonstrates stage‐specific effects of astrocytic reactivity on Alzheimer's Disease pathology

**DOI:** 10.1002/alz70856_100751

**Published:** 2025-12-25

**Authors:** Marie Emilie Tuil, Bshaier Allehyany, Paul Edison

**Affiliations:** ^1^ Imperial College London, London, United Kingdom; ^2^ Imperial College London, London, Greater London, United Kingdom; ^3^ Division of Neurology, Department of Brain Sciences, Imperial College London, United Kingdom, London, London, United Kingdom

## Abstract

**Background:**

Astrocytic and microglial reactivity play a significant role in AD pathology, yet studies show conflicting results. There is growing evidence of several microglial responses, either neuroprotective or neurotoxic^1,2^. Because of astrocyte‐microglia crosstalk^3^, we hypothesised that the astrocytic response shows similar neuroprotective and neurotoxic, and stage and region‐specific patterns in AD.

We evaluated the relationship between AD biomarkers and cognition to understand the impact of the astrocytic response on pathology throughout AD trajectory.

**Method:**

323 participants, with CSF YKL‐40, GAP‐43, Aβ_1‐42_, T‐tau, and *p*‐tau_181_, MRI, 18F‐AV45, 18F‐FDG scans, and ADAS‐Cog‐13 scores, were selected from the ADNI database (https://adni.loni.usc.edu/). They were stratified into: Aβ+AD (*n* = 89), Aβ+MCI (*n* = 140), and Aβ−MCI (*n* = 94). We evaluated the relationships between biomarkers and cognition using Pearson correlations, and between YKL‐40, GAP‐43, 18F‐AV45 and 18F‐FDG scans using voxel‐based linear regressions.

**Result:**

Both taus are very significantly correlated (*p* <0.001) with GAP43 (r>0,697 in all groups), and with YKL‐40 (r>0.430). YKL‐40 is very significantly associated (*p* <0.001) with GAP43 in Aβ+MCI and AD (r=0.428, r=0.460), and significantly so in Aβ‐MCI (r=0.308, *p* = 0.003). Additionally, YKL‐40 is very significantly associated (*p* <0.001) with Aβ_1‐42_ in Aβ‐MCI (r=0.442), and significantly so in Aβ+MCI and AD (r=0.181, *p* = 0.033; r=0.241, *p* = 0.023). 18F‐AV45_YKL‐40 voxel‐based regressions show a Braak‐like pattern, with more clusters in Aβ+MCI than Aβ‐MCI, and none in AD.

**Conclusion:**

YKL‐40 correlations suggest stage‐dependent astrocytic responses. A pro‐inflammatory Aβ‐tau‐associated response emerges first, and becomes mostly associated with tau in Aβ+MCI and AD. Additionally, synaptic dysfunction appears closely linked to tau and YKL‐40 as AD progresses, with Braak‐like patterns emerging on 18F‐AV45_YKL‐40 images VoxelStats. This suggests tau is the main neurotoxic hallmark in AD, acting in concert with amyloid and astrocytic reactivity.

**References**

1. Ahmad MH, et al. Influence of microglia and astrocyte activation in the neuroinflammatory pathogenesis of Alzheimer's disease: Rational insights for the therapeutic approaches. *J Clin Neurosci*. Jan 2019;59:6‐11.doi:10.1016/j.jocn.2018.10.034

2. Valiukas Z, et al. Microglial activation states and their implications for Alzheimer's Disease. *J Prev Alzheimers Dis*. Jan 2025;12(1):100013.doi:10.1016/j.tjpad.2024.100013

3. Jha MK, et al. Microglia‐Astrocyte Crosstalk: An Intimate Molecular Conversation. *Neuroscientist*. Jun 2019;25(3):227‐240.doi:10.1177/1073858418783959

